# Correlation Between Radiation Damage Evolution and Mechanical Properties of Fe-Irradiated Tungsten

**DOI:** 10.3390/ma19214540

**Published:** 2026-10-28

**Authors:** Bauyrzhan Rakhadilov, Moldir Bayandinova, Riza Abilkalykova, Zarina Satbayeva, Lazat Baimoldanova

**Affiliations:** 1 PlasmaScience LLP, Ust-Kamenogorsk 070000, Kazakhstan; 2 Department of Physics, Sarsen Amanzholov East Kazakhstan University, Ust-Kamenogorsk 070000, Kazakhstan

**Keywords:** tungsten, Fe-ion irradiation, radiation damage, microstructural evolution, surface roughness

## Abstract

Tungsten is a promising plasma-facing material for fusion applications; however, irradiation-induced damage can significantly affect its structural, surface, and mechanical properties. In this work, polycrystalline tungsten was irradiated with 84 MeV ^56^Fe ions at fluences of 1 × 10^15^, 5 × 10^15^, and 1 × 10^16^ ions cm^−2^. Radiation damage was evaluated using SRIM/TRIM simulations and correlated with scanning electron microscopy, X-ray diffraction, surface roughness measurements, and nanoindentation results. The calculated projected range of Fe ions was approximately 5.57 μm, while the maximum damage increased from approximately 1.38 to 13.79 dpa with increasing fluence. SEM revealed increasingly pronounced surface modifications at higher fluences. XRD confirmed preservation of the body-centered cubic α-W structure without detectable additional phases, although small non-monotonic changes in the lattice parameter and diffraction peak widths were observed. Surface roughness showed a non-monotonic dependence on fluence, reaching maximum mean values of R_a_ = 0.083 μm and R_z_ = 0.880 μm at 5 × 10^15^ ions cm^−2^. Nanoindentation showed a tendency toward lower mean hardness and elastic modulus values after irradiation, although the differences were generally comparable to the experimental scatter. Overall, the results demonstrate a complex relationship between accumulated radiation damage and the structural, surface, and nanomechanical response of tungsten.

## 1. Introduction

Tungsten is one of the most promising materials for plasma-facing components in fusion power plants due to its exceptionally high melting point, high thermal conductivity, low sputtering rate, and good resistance to intense thermal and radiation loads [1,2,3]. These properties make it a leading candidate for the fabrication of the divertor and first wall of future fusion reactors. However, during operation, tungsten is exposed to high-energy neutrons, plasma ions, and nuclear reaction products, leading to the accumulation of radiation damage, changes in microstructure, and a deterioration in the material’s operational properties.

Numerical simulation is an effective tool for studying physical processes and is widely used to analyze material behavior, predict material properties, optimize process parameters, and interpret experimental results [4,5]. Accelerated-ion irradiation is widely used to simulate radiation effects, as it allows the processes of radiation damage accumulation to be reproduced in a significantly shorter time compared to neutron irradiation [6].

Heavy-ion irradiation is of particular interest, as it is accompanied by intense cascades of atomic collisions and the formation of a high concentration of radiation defects in the near-surface layer of the material. Iron ions are widely used to simulate radiation damage in tungsten because they are characterized by significant energy losses due to nuclear stopping, leading to the formation of vacancies, interstitial atoms, dislocation loops, and defect clusters. The development of such defect structures has a significant impact on the surface morphology, crystal lattice parameters, roughness, and mechanical properties of tungsten [7,8,9,10].

In recent years, a number of studies have been published on the effect of various types of ion irradiation on the structural state of tungsten. In the work of El-Atwani et al. [11], it was shown that heavy-ion irradiation of tungsten is accompanied by the accumulation of dislocation loops and other cascade-induced defects, with the nature of their evolution depending on temperature, the dose of damage (dpa), and the initial microstructure of the material. Similar results were obtained by Klimenkov et al. [12], who, using transmission electron microscopy, demonstrated the formation of radiation-induced defect clusters and the preservation of the body-centered cubic structure of α-W even at significant irradiation doses.

In a review article by Hu [13], it is shown that the accumulation of radiation-induced defects significantly alters the mechanical behavior of tungsten, causing radiation hardening and embrittlement of the material. As the irradiation dose increases, the evolution of dislocation loops, vacancies, and precipitates leads to further changes in hardness, yield strength, and the ductile-to-brittle transition temperature. According to Zhang et al. [14], the accumulation of radiation-induced defects following Fe-ion irradiation leads to an increase in the hardness of tungsten, indicating the development of radiation hardening in the material.

A substantial number of previous studies have focused on tungsten irradiated with helium [15], hydrogen [16], argon [17], and tungsten self-ions [18,19,20]. These investigations have demonstrated that radiation-induced defect accumulation can lead to microstructural changes, irradiation hardening or softening, surface modification, and variations in mechanical properties [21,22]. However, comparatively fewer studies have systematically examined Fe-ion-irradiated tungsten using an integrated approach that combines SRIM calculations with scanning electron microscopy (SEM), X-ray diffraction (XRD), surface roughness measurements, and nanoindentation. Consequently, the quantitative relationship between the calculated damage distribution and experimentally observed changes in the structural, surface, and nanomechanical properties of tungsten remains insufficiently understood.

The aim of this work is to investigate the effects of Fe-ion irradiation on the surface morphology, crystal structure, surface roughness, and nanomechanical properties of tungsten. The ion penetration profile and depth distribution of radiation damage were calculated using the SRIM code. The simulated results were correlated with experimental data obtained by SEM, XRD, surface roughness measurements, and instrumental nanoindentation. This combined approach was used to clarify the relationship between radiation damage accumulation and changes in the structural and mechanical state of tungsten.

## 2. Materials and Methods

### 2.1. Material

The study material consisted of polycrystalline tungsten with a purity of 99.95 wt.% in the form of 10-mm-diameter discs. Prior to irradiation, the sample surfaces were sequentially ground and mechanically polished.

### 2.2. Fe-Ion Irradiation

The irradiation was performed on the DC-60 heavy-ion cyclotron (Institute of Nuclear Physics, Astana, Kazakhstan). The samples were irradiated with ^56^Fe^6+^ ions at an energy of 1.5 MeV/u at room temperature at fluences of 1 × 10^15^, 5 × 10^15^, and 1 × 10^16^ ions cm^−2^. Irradiation was conducted at room temperature as a controlled condition to minimize the contribution of thermally activated defect migration and annealing during irradiation. The iron ion beam was generated using an electron cyclotron resonance (ECR) ion source, after which the ions were accelerated in the DC-60 cyclotron’s magnetoresonance system. The acceleration parameters were preliminarily calculated using the DC-60 Calc software (version 1.0), after which the accelerator complex was tuned to obtain the required energy, intensity, and stability parameters of the ion beam.

### 2.3. SRIM Simulation

To simulate the interaction of Fe ions with tungsten, the Stopping and Range of Ions in Matter (SRIM-2013) program was used in TRIM mode. Calculations were performed at total ion energies of 74, 84, and 94 MeV, with 84 MeV corresponding to the experimental irradiation energy. The Full Cascades method was used with a displacement threshold energy of 90 eV and a tungsten atomic number density of 6.334 × 10^22^ atoms cm^−3^. A total of 10,000 ions were simulated for each energy. The projected ion range, longitudinal and transverse straggling, atomic displacements, and vacancies per incident ion were analyzed.

### 2.4. SEM

Microstructural characterization and energy-dispersive X-ray spectroscopy (EDS) of the irradiated samples were performed using an SEM3200 scanning electron microscope (CIQTEK Co., Ltd., Hefei, China) equipped with a tungsten cathode. The studies were conducted at an accelerating voltage of 15 kV in low-vacuum mode. A secondary electron (SE) detector was used to obtain the micrographs.

### 2.5. XRD

X-ray phase analysis (XRD) was performed on a D6 PHASER Material Research diffractometer (Bruker AXS GmbH, Karlsruhe, Germany) using Cu Kα radiation (λ = 0.15406 nm) at 40 kV and 15 mA. Diffractograms were recorded in the range 2θ = 20–90° with a step size of 0.02°. The diffraction data were analyzed using PowderCell 2.5 software to determine the lattice parameter and full width at half maximum (FWHM) values of the diffraction peaks.

### 2.6. Nanoindentation

Instrumental nanoindentation was performed on a FISCHERSCOPE HM2000 instrument (Helmut Fischer GmbH Institut für Elektronik und Messtechnik, Sindelfingen, Germany) in accordance with DIN EN ISO 14577-1 [23] using a Vickers diamond indenter. The maximum test load was 10 mN, and the loading time was 15 s; after reaching the maximum load, the specimen was held for 5 s to account for material creep. The indenter feed rate was 2 μm/s. The maximum indentation depth under the applied load of 10 mN was approximately 0.25 μm. Preliminary processing of the experimental data was performed using WIN-HCU software (version 7.1). The Martens hardness (HM), indentation hardness (Hit), the reduced modulus of elasticity EIT/(1 − ν^2^), and the Vickers hardness (HV) were obtained using the instrument software in accordance with the requirements of DIN EN ISO 14577-1. The results are presented as mean ± standard deviation (SD), with n = 6, 8, 7, and 6 valid indentations for pristine W and the samples irradiated at 1 × 10^15^, 5 × 10^15^, and 1 × 10^16^ ions cm^−2^, respectively. The maximum indentation depth (~0.25 μm) was substantially smaller than the SRIM-calculated projected range of 84 MeV Fe ions in tungsten (Rp ≈ 5.57 μm). Therefore, the nanoindentation measurements predominantly characterize the mechanical response of the Fe-ion-affected near-surface region.

### 2.7. Surface Roughness

Surface roughness parameters were determined using a HXSRT-200A portable contact profilometer (Dandong Haoxiang Instruments Co., Ltd., Dandong, China). Measurements were taken at a cut-off length of 2.5 mm and an evaluation length of 2.5 mm, corresponding to one cut-off length. Based on the recorded surface profiles, the arithmetic mean roughness (R_a_) and the average maximum profile height (Rz) were determined. For each sample, seven independent measurements were performed at different locations on the surface. The results are presented as the mean ± standard deviation (mean ± SD, n = 7).

## 3. Results

### 3.1. SRIM Simulation

**Figure 1:**
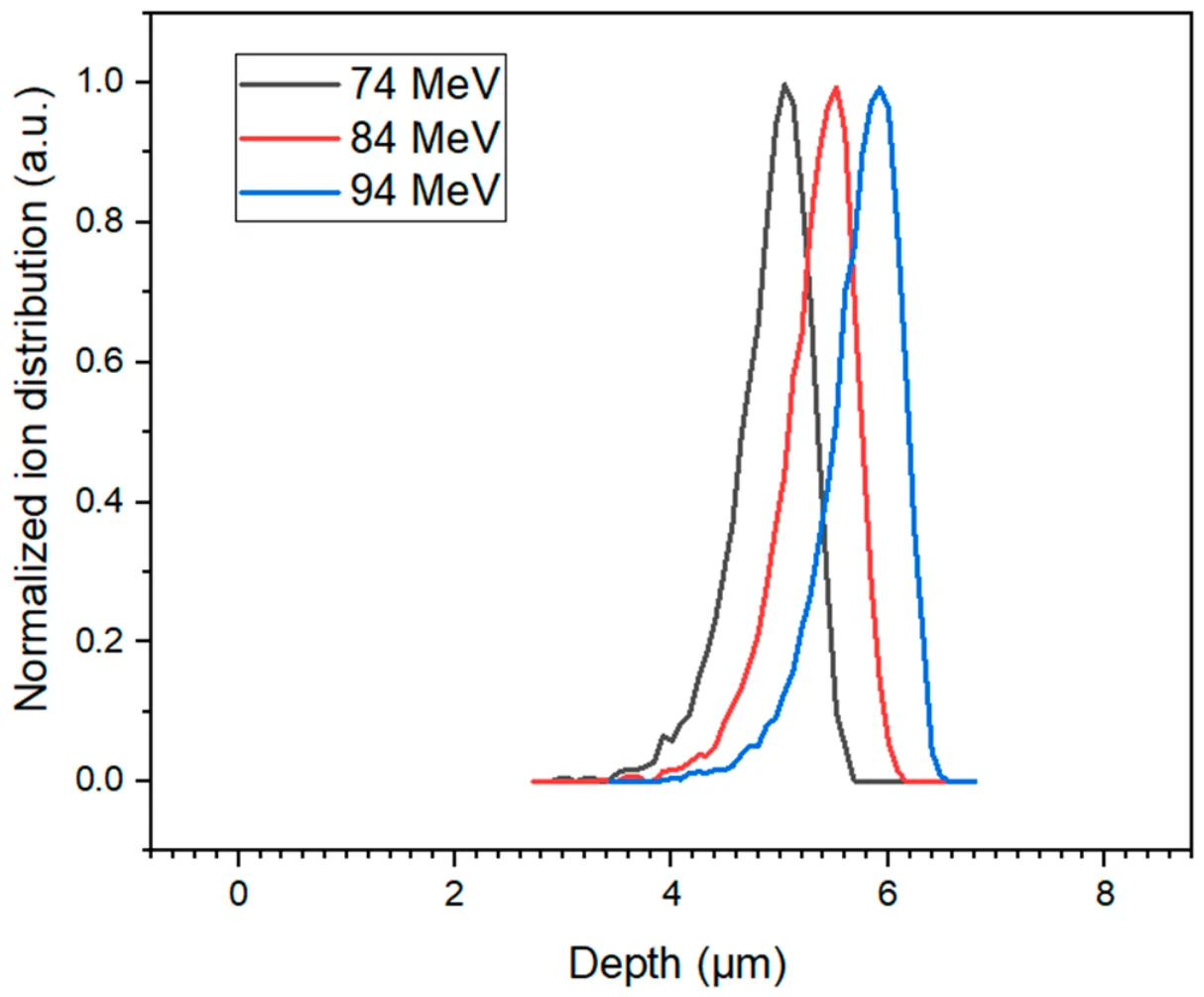
SRIM/TRIM-calculated depth distributions of ^56^Fe ions in tungsten at total ion energies of 74, 84, and 94 MeV.

Figure 1 shows the calculated depth distributions of ^56^Fe ions in tungsten at total ion energies of 74, 84, and 94 MeV. With increasing incident ion energy, the projected range R_p_ increases progressively from 5.13 μm at 74 MeV to 5.57 μm at 84 MeV and 6.00 μm at 94 MeV. At the same time, the longitudinal straggling changes only slightly, with corresponding values of 0.4374, 0.4470, and 0.4465 μm. Thus, within the investigated energy range, increasing the ion energy primarily shifts the implantation region deeper into the material without substantial broadening of the distribution.

The main calculated parameters of ion penetration and radiation damage are summarized in Table 1. The number of target atom displacements increases slightly from 13,924 displacements/ion at 74 MeV to 14,007 displacements/ion at 84 MeV and 14,117 displacements/ion at 94 MeV. A similar trend is observed for vacancy production, with 13,066, 13,216, and 13,325 vacancies/ion, respectively. These results indicate that, within the investigated energy range, the variation in ion energy has a more pronounced effect on the penetration depth than on the total number of radiation-induced defects produced by a single incident ion.

Since the ion energy was kept constant at 84 MeV in the experiments, while the irradiation fluence was varied, the atomic displacement profile calculated at 84 MeV was used to determine the damage in units of displacements per atom (dpa) for the three experimental fluences [24,25]. The depth-dependent dpa distribution was calculated from the atomic displacement profile obtained using SRIM/TRIM according to:

$$\mathrm{dpa}\left(x\right)=\;\frac{D(x)\Phi }{N_{W}\times 10^{-8}}$$

where *D(x)* is the depth-dependent density of atomic displacements calculated using SRIM/TRIM, in displacements/(Å·ion); *Φ* is the irradiation fluence, in ions/cm^2^; *N_W_* = 6.334 × 10^22^ atoms/cm^3^ is the atomic number density of tungsten; and the factor 10^−8^ accounts for the conversion from Å to cm. The depth profile of the total atomic displacements was determined as

*D*(*x*) = *V*(*x*) + *R*(*x*)

where *V(x)* is the depth distribution of vacancies and *R(x)* is the depth distribution of replacement collisions calculated using SRIM/TRIM. The resulting total atomic displacement profile was used to calculate the dpa values for the three experimental fluences.

**Figure 2:**
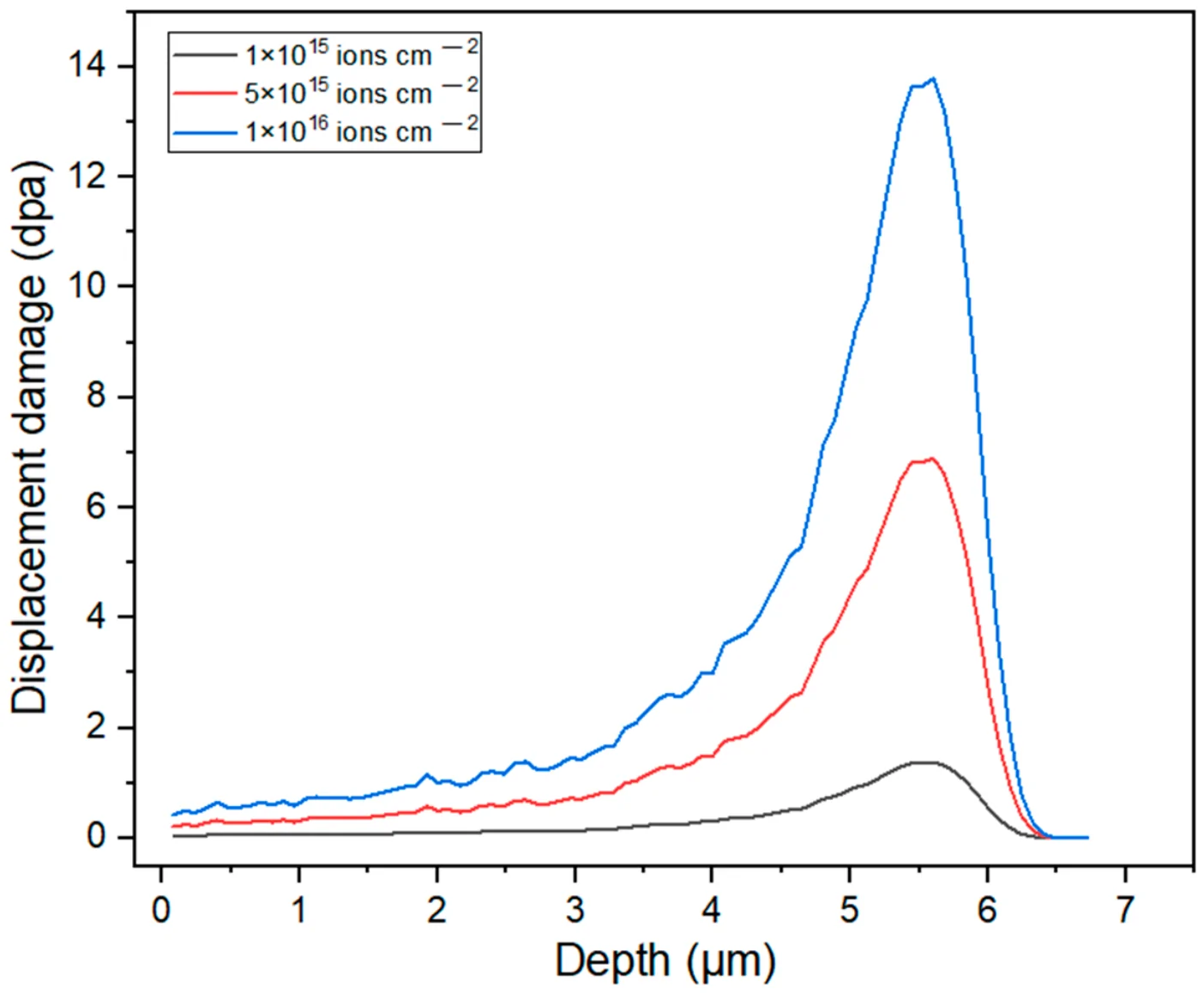
Depth distribution of displacement damage (dpa) in tungsten under 84 MeV ^56^Fe ion irradiation at fluences of 1 × 10^15^, 5 × 10^15^ and 1 × 10^16^ ions/cm^2^.

Figure 2 shows the calculated depth-dependent dpa profiles for the experimental fluences of 1 × 10^15^, 5 × 10^15^ and 1 × 10^16^ ions/cm^2^. Since the incident ion energy was identical in all experiments, the position of the damage maximum and the shape of the damage profile remain essentially unchanged, whereas the calculated damage increases proportionally with fluence. The damage maximum is located at a depth of approximately 5.60 μm, close to the calculated projected ion range of R_p_ = 5.57 μm. The maximum damage values are approximately 1.38, 6.89, and 13.79 dpa for fluences of 1 × 10^15^, 5 × 10^15^ and 1 × 10^16^ ions/cm^2^ respectively.

The obtained damage profiles enable a quantitative comparison of the calculated radiation damage level with the changes in the structural, surface, and nanomechanical characteristics of tungsten discussed in the following sections. The calculations at 74 and 94 MeV were performed only to evaluate the effect of ion energy on the penetration and defect formation characteristics relative to the experimental condition, whereas the dpa calculations for the three experimental fluences were based on the damage profile at the actual irradiation energy of 84 MeV.

### 3.2. SEM

**Figure 3:**
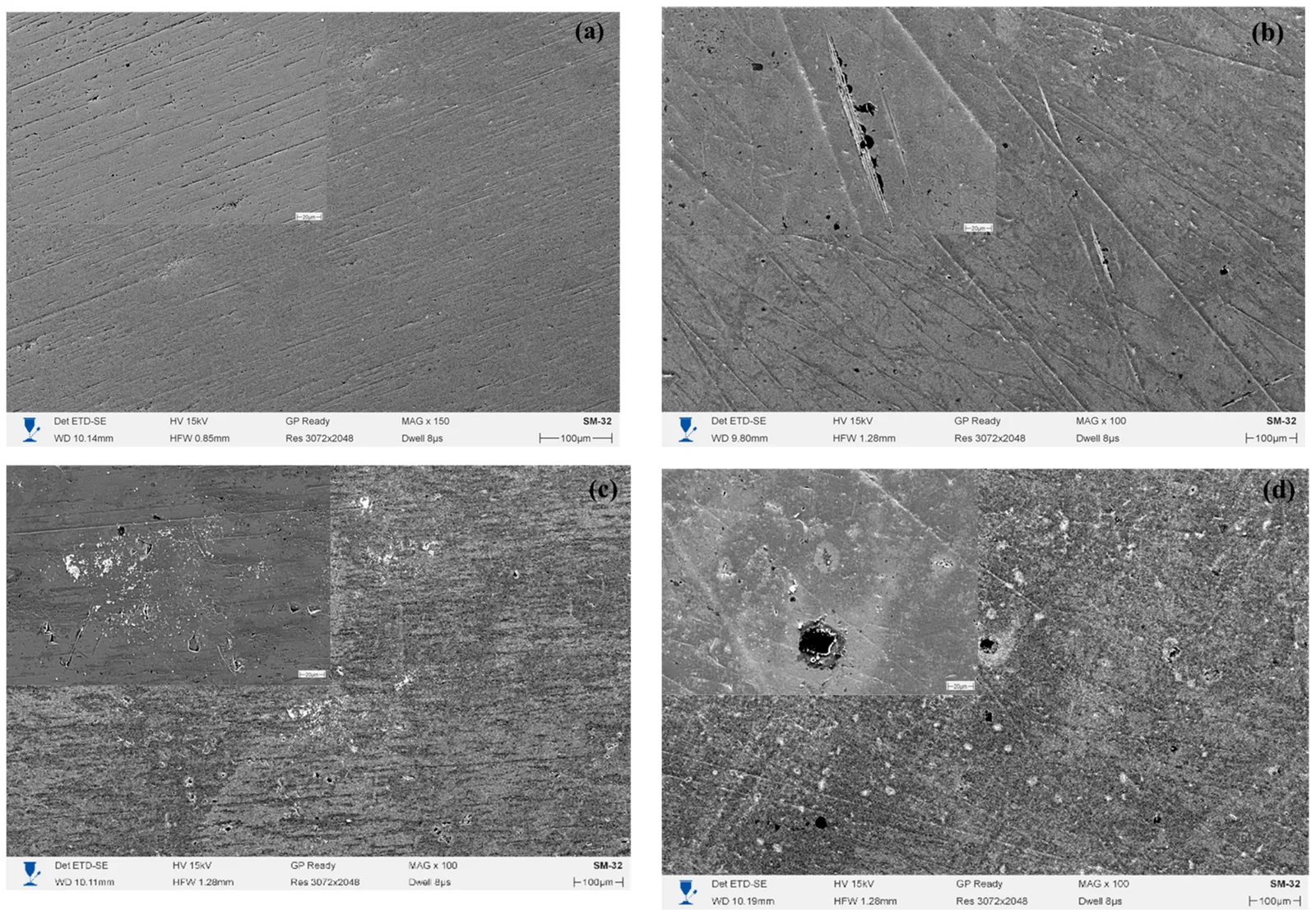
SEM micrographs of pristine tungsten and Fe-ion-irradiated samples: (**a**) pristine W; (**b**) 1 × 10^15^; (**c**) 5 × 10^15^; and (**d**) 1 × 10^16^ ions cm^−2^.

Figure 3 shows SEM images of the as-received tungsten and samples irradiated with Fe ions at fluences of 1 × 10^15^, 5 × 10^15^, and 1 × 10^16^ ions cm^−2^. The as-received tungsten (Figure 3a) is characterized by a relatively homogeneous surface with clearly distinguishable traces of mechanical processing. There are no large pores or cracks. After irradiation at a fluence of 1 × 10^15^ ions cm^−2^ (Figure 3b), the surface morphology is generally preserved; however, isolated local depressions and inhomogeneities appear, which may be associated with the effects of ion irradiation. As the fluence increases to 5 × 10^15^ ions cm^−2^ (Figure 3c), surface inhomogeneity increases, localized light-colored areas appear, and the number of surface defects increases. The sample irradiated at 1 × 10^16^ ions cm^−2^ (Figure 3d) exhibits the most pronounced changes: isolated pores, depressions, and a more developed heterogeneous structure are observed. The observed changes in surface morphology become more pronounced with increasing irradiation fluence and may be associated with the accumulation of irradiation-induced defects and the overlap of atomic displacement cascades. The observed surface evolution is consistent with the work of El-Atwani et al. [11], Klimenkov et al. [12], and Yi et al. [26], in which a similar increase in surface morphology complexity and development of the defect structure in tungsten were observed with increasing ion irradiation dose.

**Figure 4:**
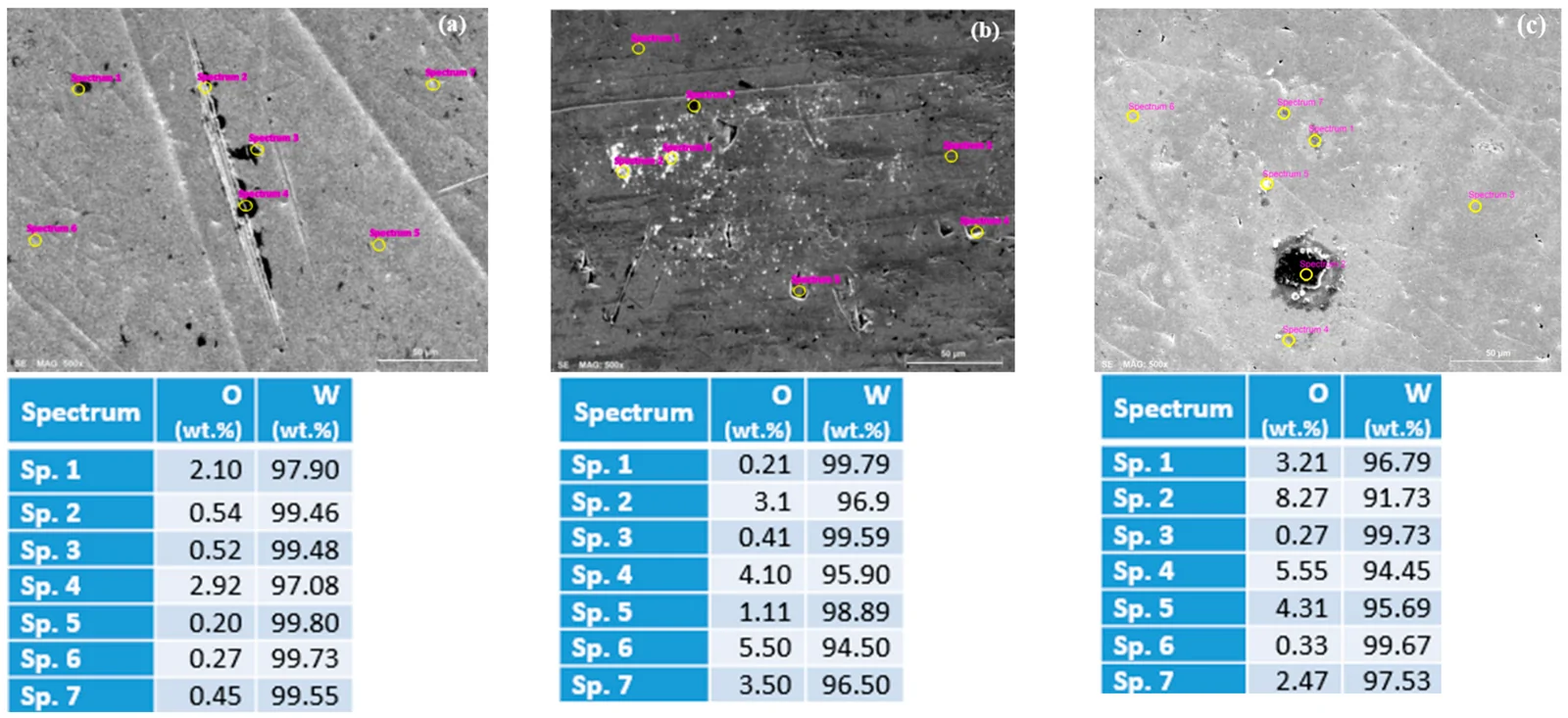
SEM micrographs showing the locations of point EDS measurements and the corresponding elemental compositions of Fe-ion-irradiated tungsten: (**a**) 1 × 10^15^; (**b**) 5 × 10^15^; and (**c**) 1 × 10^16^ ions cm^−2^.

Point EDS analysis was performed to determine the local elemental composition of characteristic surface regions (Figure 4). For the sample irradiated at a fluence of 1 × 10^15^ ions cm^−2^, all examined regions were characterized by a high W content (97.08–99.80 wt.%) and a low O content (0.20–2.92 wt.%). At a fluence of 5 × 10^15^ ions cm^−2^, the oxygen content at the analyzed points ranged from 0.21 to 5.50 wt.%, while the W content ranged from 94.50 to 99.79 wt.%. For the sample irradiated at 1 × 10^16^ ions cm^−2^, the oxygen content varied from 0.27 to 8.27 wt.%, while most of the analyzed regions retained a high W content.

The observed variation in oxygen content between individual measurement points indicates its heterogeneous distribution over the surface. Since irradiation was performed under vacuum and the samples were subsequently exposed to ambient air prior to SEM/EDS examination, the detected oxygen may at least partly originate from post-irradiation surface oxidation and therefore cannot be attributed exclusively to ion irradiation. In addition, the oxygen concentrations determined by EDS should be considered semi-quantitative.

According to calculations by Alkhamees et al. [27], oxygen atoms can be effectively trapped by vacancies in tungsten to form stable O–V complexes. Hu et al. [28] also reported that oxygen can interact with irradiation-induced vacancies in tungsten following 2.5 MeV electron irradiation, contributing to the formation of O–V complexes and defect evolution. Although their irradiation conditions differed from the present Fe-ion irradiation study, their results support the possible role of oxygen in radiation defect evolution. Thus, previous studies indicate a possible interaction between oxygen and irradiation-induced defects in tungsten; however, the EDS results obtained in the present study alone do not allow the mechanism responsible for the presence of oxygen on the surface to be unambiguously determined.

### 3.3. XRD

**Figure 5:**
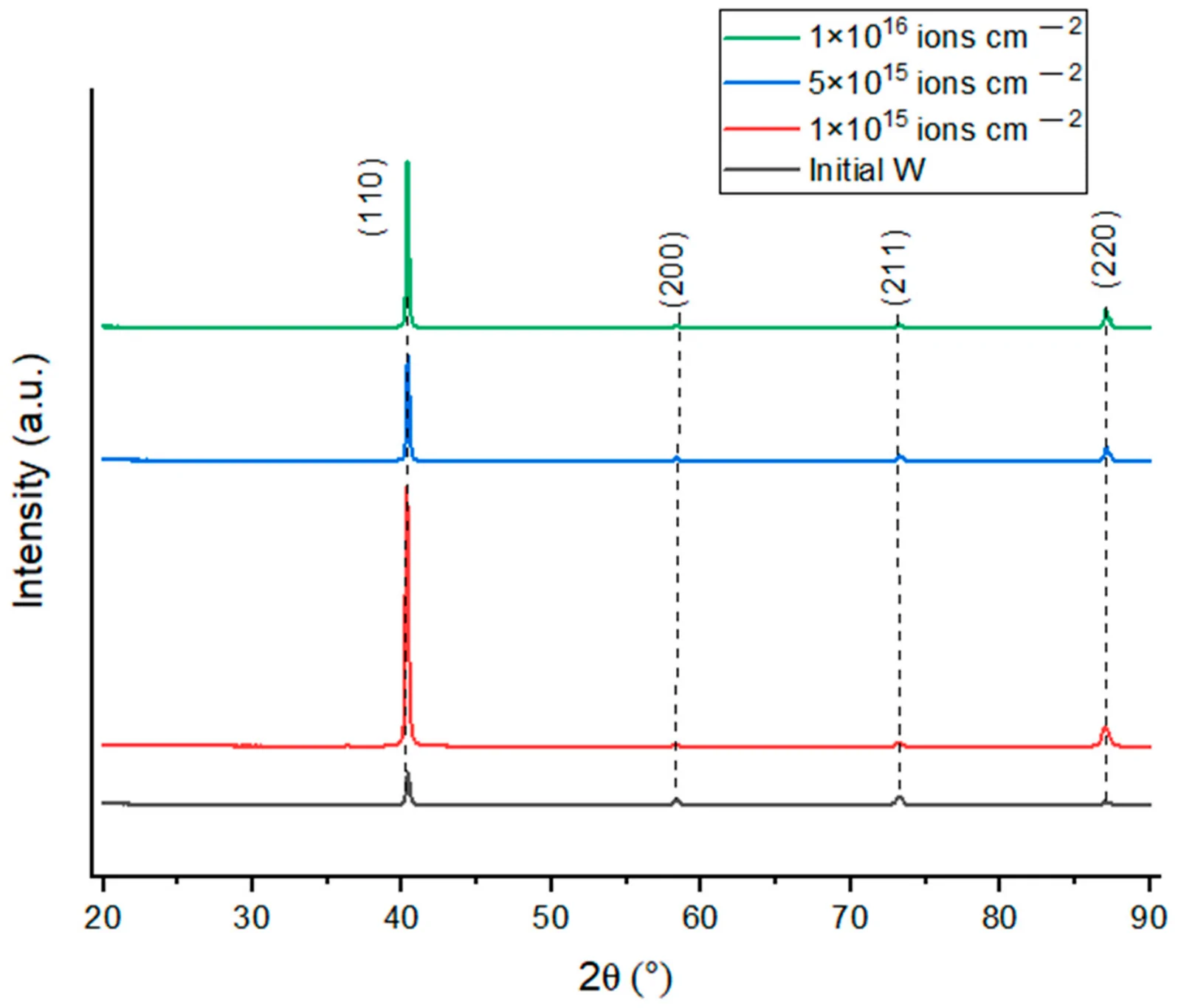
XRD patterns of pristine and Fe-ion-irradiated tungsten at different irradiation fluences.

Figure 5 shows X-ray diffraction patterns of the as-received tungsten and samples irradiated with Fe ions at fluences of 1 × 10^15^, 5 × 10^15^, and 1 × 10^16^ ions cm^−2^. In all states studied, diffraction peaks are observed at 2θ ≈ 40.3°, 58.3°, 73.2°, and 87.0°, corresponding to the (110), (200), (211), and (220) reflections of the body-centered cubic α-W structure (ICDD PDF No. 04-0806). The (110) reflection is the most intense, which is due to the texture of the starting material. After irradiation, no additional diffraction peaks corresponding to intermetallic phases of the W–Fe system or tungsten oxides were detected within the sensitivity limits of the method.

For a quantitative evaluation of changes in the crystal structure, the lattice parameter *a* and the full width at half maximum (FWHM) of the diffraction peaks were determined. The lattice parameter of the pristine tungsten was 3.1701 Å. After Fe-ion irradiation at fluences of 1 × 10^15^, 5 × 10^15^, and 1 × 10^16^ ions cm^−2^, the lattice parameter values were 3.1688, 3.1648, and 3.1692 Å, respectively. Thus, small and non-monotonic changes in the lattice parameter were observed within the investigated fluence range.

The FWHM values of the α-W reflections are presented in Table 2. For pristine tungsten, the FWHM values of the (110), (200), (211), and (220) reflections were 0.315°, 0.344°, 0.345°, and 0.314°, respectively. Changes in the diffraction peak widths were observed after irradiation. For the (200), (211), and (220) reflections, the FWHM values of the irradiated samples were higher than those of pristine tungsten, whereas the (110) reflection showed a decrease in FWHM. Moreover, the FWHM values of the (200), (211), and (220) reflections exhibited a non-monotonic dependence on irradiation fluence.

The different FWHM responses of the individual reflections may reflect an anisotropic response of the crystal lattice to irradiation-induced defects. In particular, the stronger broadening of the (220) reflection compared with the (110) reflection may be associated with the different sensitivity of these reflections to irradiation-induced lattice strain and local lattice distortions. However, the present XRD data do not allow specific defect types to be assigned to individual crystallographic planes.

Thus, Fe-ion irradiation within the investigated fluence range does not lead to detectable phase transformations, and the body-centered cubic α-W structure is preserved. At the same time, the observed changes in the lattice parameter and FWHM indicate that the characteristics of the diffraction reflections change after irradiation. The obtained results are in good agreement with the data reported by Klimenkov et al. [12] and Habainy J. et al. [29], who also demonstrated the preservation of the α-W structure under irradiation.

### 3.4. Nanoindentation Analysis

**Figure 6:**
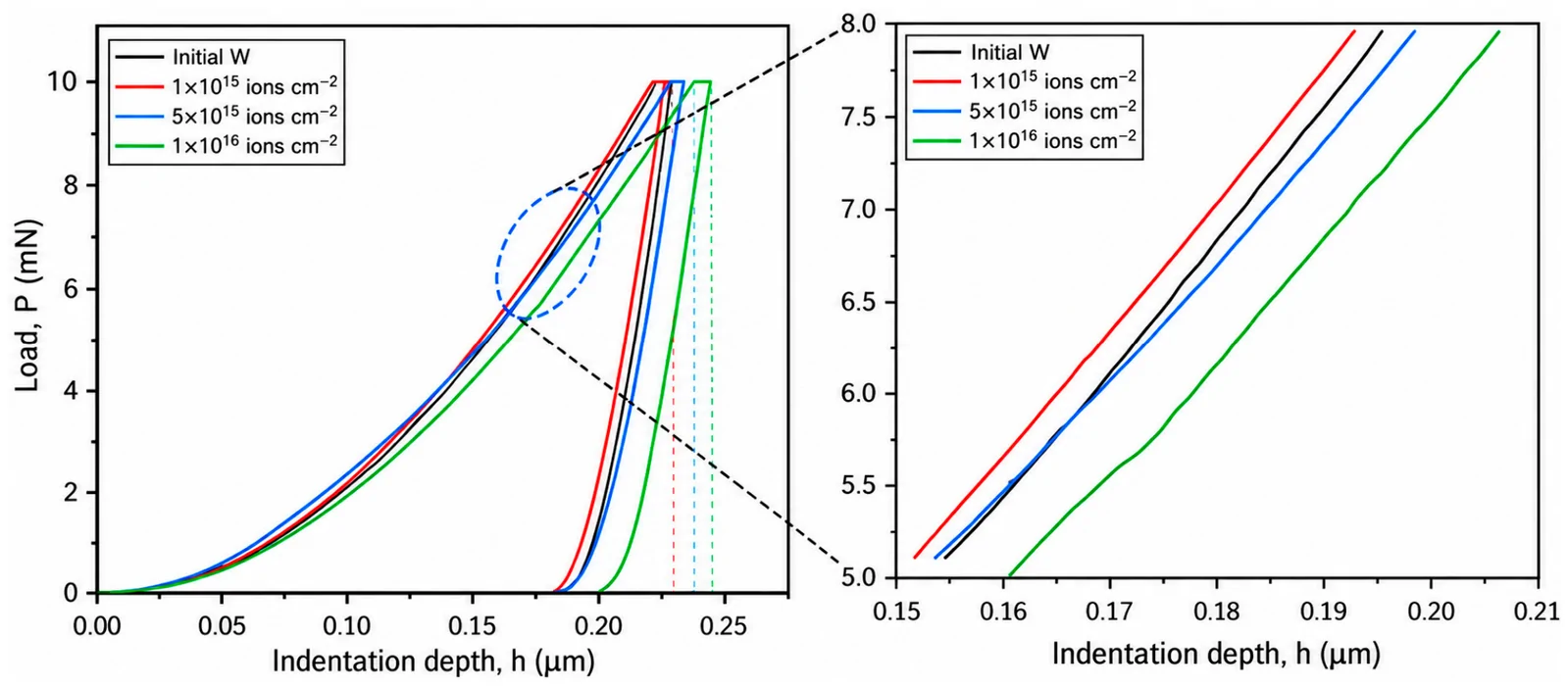
Nanoindentation load–displacement curves of pristine tungsten and Fe-ion-irradiated tungsten at fluences of 1 × 10^15^, 5 × 10^15^, and 1 × 10^16^ ions cm^−2^. The inset shows an enlarged view of the loading segment, highlighting differences in the indentation response.

Figure 6 shows the “load–penetration depth” (P–h) curves obtained from nanoindentation of pristine tungsten and samples irradiated with Fe ions at fluences of 1 × 10^15^, 5 × 10^15^, and 1 × 10^16^ ions cm^−2^. The load curves for all samples are similar in nature; however, slight differences in the indenter penetration depth are observed. For the Fe 1 × 10^15^ and Fe 5 × 10^15^ samples, the penetration depth is slightly less than that of the Fe 1 × 10^16^ sample, while the unirradiated tungsten occupies an intermediate position. These differences suggest changes in the material’s mechanical response following ion irradiation, which were further evaluated using the nanoindentation-derived mechanical parameters.

The nanoindentation-derived mechanical properties are summarized in Table 3. After irradiation at a fluence of 1 × 10^15^ ions cm^−2^, the Martens hardness (HM) was 7003.8 ± 437.4 N/mm^2^, and the elastic modulus (EIT) was 426.0 ± 20.1 GPa. As the fluence increased to 5 × 10^15^ ions cm^−2^, the HM value rose slightly to 7180.8 ± 640.7 N/mm^2^, but remained below that of the as-received material. At a maximum fluence of 1 × 10^16^ ions cm^−2^, the mean HM value decreased to 6632.2 ± 480.5 N/mm^2^, while the mean values of HV and Hit were 811.2 ± 65.6 and 8583.9 ± 694.4 N/mm^2^, respectively. The mean nanoindentation parameters showed a tendency to decrease after irradiation; however, the differences were generally comparable to the experimental scatter.

The SRIM simulation data indicate the formation of a significant number of radiation-induced defects in the near-surface region. The observed changes in the nanoindentation parameters may be associated with the evolution of this defect structure. Unlike the radiation hardening reported by Zhang et al. [19], the present results show only a tendency toward lower mean HM, HV, and Hit values after Fe-ion irradiation, with differences generally comparable to the experimental scatter. Similar studies show that changes in the mechanical properties of irradiated tungsten depend significantly on the irradiation conditions, test temperature, material microstructure, and depth of the damaged layer [30,31]. These differences are likely due to the specific irradiation conditions and the evolution of the defect structure in tungsten.

### 3.5. Surface Roughness Analysis

**Figure 7:**
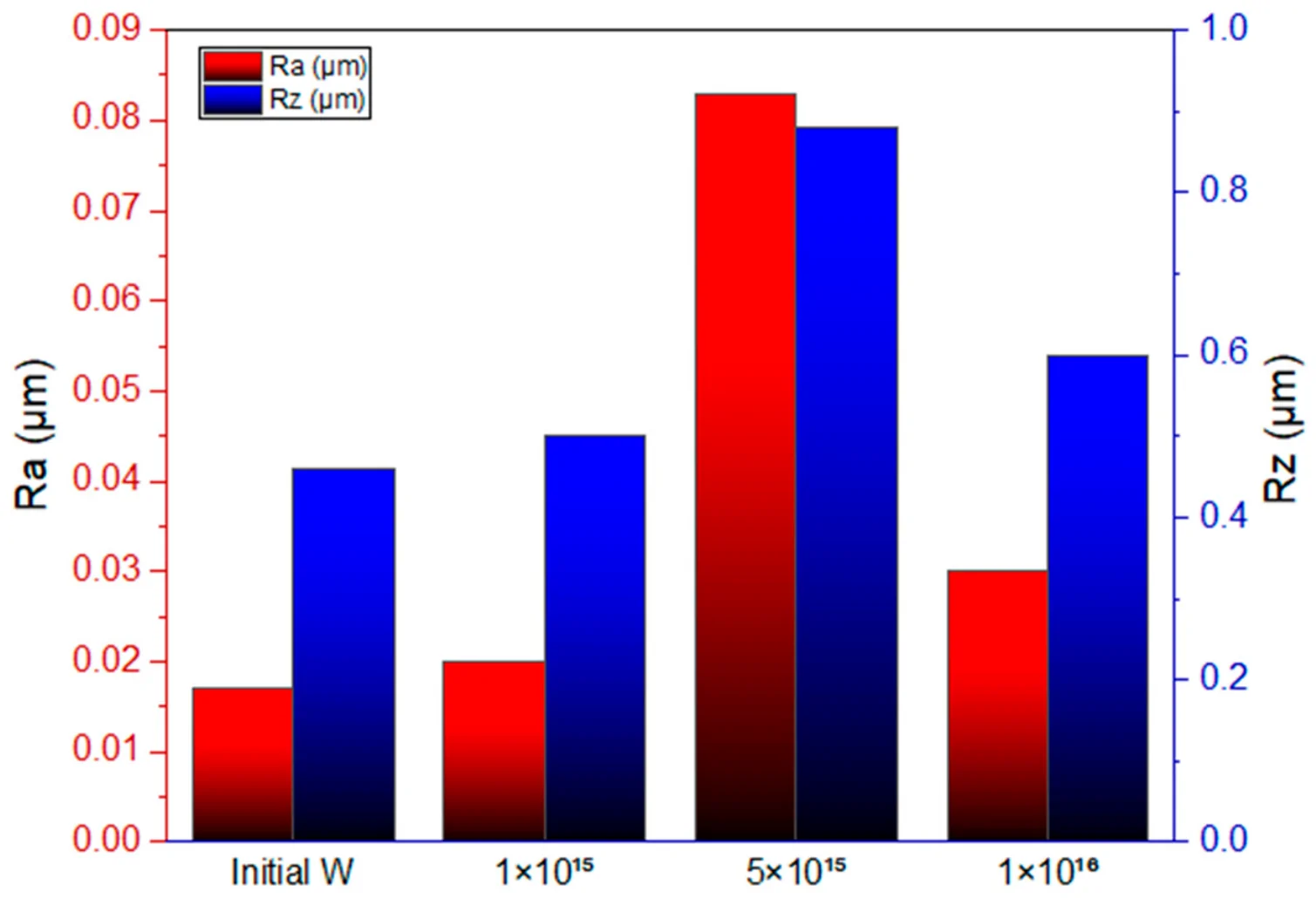
Evolution of the surface roughness parameters R_a_ and R_z_ of tungsten as a function of Fe-ion fluence.

The surface roughness results for pristine tungsten and samples irradiated with Fe ions at different fluences are presented in Figure 7 and Table 4. The R_a_ and R_z_ parameters were analyzed to evaluate changes in the surface condition.

For the pristine sample, R_a_ and R_z_ were 0.017 ± 0.006 μm and 0.460 ± 0.010 μm, respectively. After irradiation at a fluence of 1 × 10^15^ ions cm^−2^, the values were R_a_ = 0.020 ± 0.017 μm and R_z_ = 0.500 ± 0.106 μm. The highest mean values were observed at 5 × 10^15^ ions cm^−2^, with R_a_ = 0.083 ± 0.006 μm and R_z_ = 0.880 ± 0.070 μm. At the highest fluence of 1 × 10^16^ ions cm^−2^, the values decreased to R_a_ = 0.030 ± 0.026 μm and R_z_ = 0.600 ± 0.257 μm.

Comparison of the SEM and profilometry results shows that both methods reveal irradiation-induced surface modifications, although the observed changes do not follow exactly the same trend with fluence. SEM provides information on local morphological features, whereas contact profilometry characterizes surface height variations over a longer profile (2.5 mm in the present study) [32]. Thus, local pores and depressions visible in the SEM images may not directly correspond to the mean R_a_ and R_z_ values, which can explain the differences between the trends observed by the two methods.

The decrease in R_a_ and R_z_ at 1 × 10^16^ ions cm^−2^ does not contradict the increase in defect production predicted by the SRIM simulations. SRIM characterizes irradiation-induced defect production within the irradiated layer, whereas R_a_ and R_z_ characterize the geometrical topography of the surface. Therefore, an increase in defect production does not necessarily result in a monotonic increase in surface roughness. The observed decrease in roughness at the highest fluence may reflect the interplay of irradiation-induced surface modification processes, such as sputtering, redeposition, and the evolution of surface morphology. Previous studies have demonstrated a close relationship between surface roughness, sputtering, and redeposition processes in ion-irradiated tungsten [33,34].

Thus, Fe-ion irradiation leads to non-monotonic changes in the surface roughness of tungsten. The highest mean R_a_ and R_z_ values were observed at a fluence of 5 × 10^15^ ions cm^−2^, followed by a decrease at 1 × 10^16^ ions cm^−2^. This behavior indicates that the evolution of surface topography does not directly follow the accumulation of irradiation-induced defects and may reflect the contribution of competing surface modification processes at high fluence.

## 4. Conclusions

This study investigated the relationship between radiation damage accumulation and changes in the structural, surface, and nanomechanical properties of tungsten irradiated with 84 MeV ^56^Fe ions at fluences from 1 × 10^15^ to 1 × 10^16^ ions cm^−2^. The main conclusions are as follows:

- SRIM/TRIM simulations showed a projected Fe-ion range of approximately 5.57 μm at the experimental energy of 84 MeV. The calculated maximum damage increased proportionally with fluence from approximately 1.38 dpa at 1 × 10^15^ ions cm^−2^ to 13.79 dpa at 1 × 10^16^ ions cm^−2^, while the position of the damage maximum remained near 5.6 μm.
- SEM observations revealed increasing modification and heterogeneity of the tungsten surface with increasing irradiation fluence. Point EDS analysis showed predominantly W-rich regions with locally varying oxygen contents; however, the detected oxygen cannot be attributed exclusively to irradiation because post-irradiation oxidation during exposure to ambient air may contribute to its presence.
- XRD analysis confirmed that the body-centered cubic α-W structure was preserved over the entire investigated fluence range, with no detectable additional phases. Irradiation produced only small, non-monotonic changes in the lattice parameter and changes in the FWHM of the diffraction reflections.
- Nanoindentation measurements showed a tendency toward lower mean HM, HV, Hit, and elastic modulus values after Fe-ion irradiation. However, the magnitude of these changes was generally comparable to the experimental scatter; therefore, they should be interpreted as trends rather than evidence of pronounced irradiation-induced hardening or softening.
- Surface roughness exhibited a non-monotonic dependence on irradiation fluence. The highest mean values, R_a_ = 0.083 μm and R_z_ = 0.880 μm, were obtained at 5 × 10^15^ ions cm^−2^, followed by a decrease at 1 × 10^16^ ions cm^−2^ despite the higher calculated radiation damage.

Overall, the results show that Fe-ion irradiation is accompanied by changes in surface morphology, diffraction characteristics, surface roughness, and nanomechanical response. However, these changes do not exhibit a simple monotonic relationship with the calculated dpa, and the changes in the nanoindentation parameters are generally comparable to the experimental scatter.

## Figures and Tables

**Table 1:** SRIM/TRIM-calculated parameters of ^56^Fe ion penetration and radiation damage in tungsten.

Total Ion Energy, MeV	Rp, μm	Longitudinal Straggling, μm	Displacements, Displacements/Ion	Vacancies, Vacancies/Ion
74	5.13	0.4374	13,924	13,066
84	5.57	0.4470	14,007	13,216
94	6.00	0.4465	14,117	13,325

**Table 2:** Lattice parameter and FWHM values of pristine and Fe-ion-irradiated tungsten.

Sample	Lattice Parameter, *a* (Å)	FWHM (110), °	FWHM (200), °	FWHM (211), °	FWHM (220), °
Pristine W	3.1701	0.315	0.344	0.345	0.314
Fe, 1 × 10^15^ ions cm^−2^	3.1688	0.235	0.629	0.872	1.099
Fe, 5 × 10^15^ ions cm^−2^	3.1648	0.226	0.534	0.691	0.807
Fe, 1 × 10^16^ ions cm^−2^	3.1692	0.197	0.598	0.837	1.060

**Table 3:** Nanoindentation-derived mechanical properties of pristine and Fe-ion-irradiated tungsten.

Sample	HM, N/mm^2^	EIT, GPa	HV	Hit, N/mm^2^
Initial W	7516.6 ± 574.2	457.5 ± 44.1	932.8 ± 71.1	9870.8 ± 748.1
Fe 1 × 10^15^ ions cm^−2^	7003.8 ± 437.4	426.0 ± 20.1	865.1 ± 68.3	9154.4 ± 722.5
Fe 5 × 10^15^ ions cm^−2^	7180.8 ± 640.7	391.8 ± 34.0	915.6 ± 73.7	9689.0 ± 775.3
Fe 1 × 10^16^ ions cm^−2^	6632.2 ± 480.5	396.5 ± 29.8	811.2 ± 65.6	8583.9 ± 694.4

**Table 4:** Surface roughness parameters of pristine and Fe-ion-irradiated tungsten.

Sample	R_a_, μm	R_z_, μm
Initial W	0.017 ± 0.006	0.460 ± 0.010
1 × 10^15^	0.020 ± 0.017	0.500 ± 0.106
5 × 10^15^	0.083 ± 0.006	0.880 ± 0.070
1 × 10^16^	0.030 ± 0.026	0.600 ± 0.257

## Data Availability

The original contributions presented in this study are included in the article. Further inquiries can be directed to the corresponding author.
